# The Glycerate and Phosphorylated Pathways of Serine Synthesis in Plants: The Branches of Plant Glycolysis Linking Carbon and Nitrogen Metabolism

**DOI:** 10.3389/fpls.2018.00318

**Published:** 2018-03-14

**Authors:** Abir U. Igamberdiev, Leszek A. Kleczkowski

**Affiliations:** ^1^Department of Biology, Memorial University of Newfoundland, St. John’s, NL, Canada; ^2^Department of Plant Physiology, Umeå Plant Science Centre, Umeå University, Umeå, Sweden

**Keywords:** glycerate serine pathway, phosphorylated serine pathway, γ-aminobutyric acid (GABA), plastid, glycolysis

## Abstract

Serine metabolism in plants has been studied mostly in relation to photorespiration where serine is formed from two molecules of glycine. However, two other pathways of serine formation operate in plants and represent the branches of glycolysis diverging at the level of 3-phosphoglyceric acid. One branch (the glycerate – serine pathway) is initiated in the cytosol and involves glycerate formation from 3-phosphoglycerate, while the other (the phosphorylated serine pathway) operates in plastids and forms phosphohydroxypyruvate as an intermediate. Serine formed in these pathways becomes a precursor of glycine, formate and glycolate accumulating in stress conditions. The pathways can be linked to GABA shunt via transamination reactions and via participation of the same reductase for both glyoxylate and succinic semialdehyde. In this review paper we present a hypothesis of the regulation of redox balance in stressed plant cells via participation of the reactions associated with glycerate and phosphorylated serine pathways. We consider these pathways as important processes linking carbon and nitrogen metabolism and maintaining cellular redox and energy levels in stress conditions.

## Introduction

The most studied in plants is the pathway of serine synthesis related to photorespiration. It leads to the formation of bulk amounts of serine reaching in photosynthetic cells of C3 plants concentrations of the order of tens millimolar, e.g., in cytosol of illuminated spinach leaf the reported concentration is 7.5 mM and in stroma – 4.3 mM (Winter et al., 1994), in cytosol of illuminated barley leaf – 50 mM, in stroma – 20 mM decreasing by more than twofold in darkness (Winter et al., 1993), and in phloem sap of barley – 25 mM in the light and 20 mM in darkness (Winter et al., 1992), which is the highest concentration of amino acid except glutamate and aspartate. However, the phosphorespiratory pathway of serine formation can be active only in the tissues exhibiting high photorespiration, i.e., in photosynthetic cells of C_3_ plants.

There are two more pathways of serine synthesis which are not related to photorespiration but instead related to glycolysis (Kleczkowski and Givan, 1988; Ros et al., 2013, 2014). One pathway (the glycerate-serine/non-phosphorylated pathway) is derived from PGA formed in glycolysis in the cytosol and the other (the phosphorylated pathway) occurs in plastids where it represents a deviation from plastid glycolysis. The phosphorylated pathway of serine synthesis was not studied extensively in plants until recent time, and the glycerate pathway is even less explored. The opinion exists that these are minor pathways; however, they represent the only ways of serine formation in non-photosynthetic tissues, in darkness, and in the plants exhibiting low or no photorespiration (C_4_ plants). It was demonstrated by Servaites and Ogren (1977) that a half of the rate of initial serine synthesis remained after chemical inhibition of the glycolate pathway. With the decrease of photorespiration followed by increase in anthropogenic CO_2_ (Sharkey, 1988), the role of non-photorespiratory pathways of serine biosynthesis may increase also in C_3_ plants.

It has been suggested that the non-photorespiratory pathways are activated under environmental stresses (e.g., salinity or hypoxia) and that they may be considered as alternatives to glycolysis where glycine is produced from serine and then glycolate is formed, with the whole pathways contributing to the redox status during stress (Ho and Saito, 2001; Ros et al., 2014). The pool of serine formed in these pathways may have a connection with the shunt of γ-aminobutyric acid (GABA) (Anoman et al., 2015), glyoxylate/formate metabolism (Igamberdiev et al., 1999), and the level of the stress hormone abscisic acid (Muñoz-Bertomeu et al., 2011). In this paper we discuss the two variations of the glycolytic metabolism that result in serine formation and link carbon and nitrogen metabolism in plant cells. The role of consequent serine conversions is analyzed from the point of regulation of redox balance in plants in the conditions of abiotic stress.

## Non-Phosphorylated (Glycerate) Serine Pathway

### Overview

**FIGURE 1:**
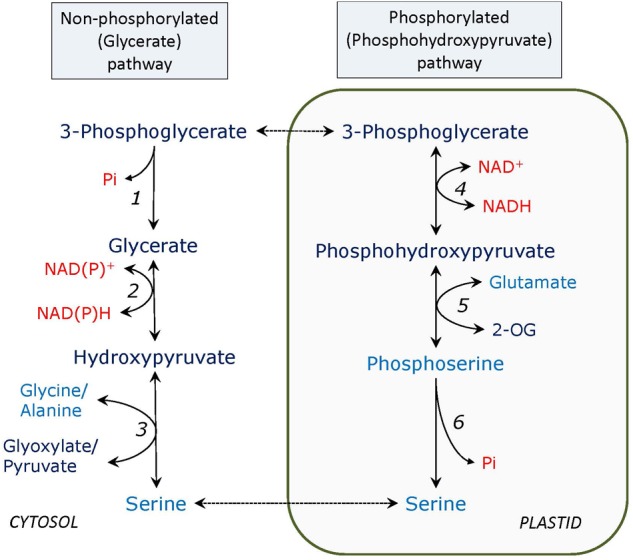
Non-phosphorylated (glycerate) and phosphorylated (phosphohydroxypyruvate) pathways of serine formation. Enzymes: 1, PGA phosphatase; 2, glycerate dehydrogenase; 3, serine: glyoxylate and serine: pyruvate aminotransferase; 4, PGA dehydrogenase; 5, phosphoserine: 2-oxogutarate aminotransferase; 6, phosphoserine phosphatase. 2-OG – 2-oxoglutarate. In all figures amino acids are depicted in blue, adenylates, phosphate, and pyridine nucleotides – in red.

The glycerate – serine non-phosphorylated pathway (**Figure 1**) can be considered as the reversal of the second half of the photorespiratory glycolate pathway, called glycerate pathway, which starts from serine and yields 3-phosphoglycerate (PGA); however, generally this is not correct. It seems that only one enzyme is different - 3-phosphoglycerate phosphatase (PGA phosphatase) instead of glycerate kinase in photorespiration, but glycerate dehydrogenase of the glycerate serine pathway is not necessarily the peroxisomal NADH-hydroxypyruvate reductase operating in reverse direction. At least this does not apply to heterotrophic tissues where photorespiratory enzymes are not expressed or exhibit low activity. The importance of this pathway has not yet been studied in detail, however, it may represent a major source of serine in photosynthetic tissues of C_4_ plants and in darkness in C_3_ plants as well as in non-photosynthetic tissues. In C_4_ plants, where photorespiration is suppressed and the photorespiratory pathway of serine formation has a very limited capacity, activities of PGA phosphatase (Randall et al., 1971), and the putative glycerate dehydrogenase (most likely corresponding to both peroxisomal HPR-1 and cytosolic HPR-2), are comparable to those in C_3_ plants (Kleczkowski and Edwards, 1989; Ueno et al., 2005).

### PGA Phosphatase

The glycerate – serine pathway is initiated in the cytosol by the enzyme PGA phosphatase. This enzyme was characterized by Randall and Tolbert (1971) and after that it was not studied extensively in plants. PGA phosphatase (EC 3.1.3.38) has a broad specificity but 3-PGA is a preferred substrate. 2-Phosphoglycolate can be also taken by this enzyme, however, the chloroplastic 2-phosphoglycolate phosphatase (EC 3.1.3.18) is specific to its substrate, and, contrary to PGA phosphatase, requires divalent cation for activity and tricarboxylic acid for stability (Randall and Tolbert, 1971). The enzyme is entirely confined to the cytosol, whereas a substantial 3-PGA-dependent phosphatase activity in chloroplast preparations has been ascribed to a contamination by a non-specific acid phosphatase (Robinson, 1982). PGA phosphatase is active at low pH (optimum pH 5.9), whereas above pH 7.5 it becomes unstable (Randall and Tolbert, 1971). Its activity was found high in a C_4_ plant (sorghum), where serine formation via the photorespiratory pathway is very limited due to a low or absent photorespiration. In this plant, its activity is fourfold higher than that of phosphoglycolate phosphatase. In sugarcane leaves, PGA phosphatase exhibited a diurnal variation increasing during late daylight hours and early evening. Its activity may be necessary for carbon transport between the bundle sheath and mesophyll cells during C_4_-photosynthesis. In the light in C_4_ plants, this enzyme is usually more active than in C_3_ plants (Randall et al., 1971).

### Glycerate Dehydrogenase/Hydroxypyruvate Reductase

The second step of the glycerate pathway of serine synthesis can be linked to isoforms of glycerate dehydrogenase/ hydroxypyruvate reductases (HPRs). These enzymes are present in the peroxisomes (HPR-1), the cytosol (HPR-2) (Kleczkowski and Randall, 1988; Kleczkowski et al., 1988, 1990; Kleczkowski and Edwards, 1989; Timm et al., 2008), and also in plastids (HPR-3) (Kleczkowski et al., 1988; Timm et al., 2011). The peroxisomal NADH-dependent hydroxypyruvate reductase (HPR-1), one of the most active photorespiratory enzymes, can also readily operate in the direction of hydroxypyruvate and serine synthesis, e.g., in darkness. This has been shown by Liang et al. (1984); in their experiments, the isolated peroxisomes from spinach readily formed serine from glycerate in the presence of NAD^+^, the amino group donor could be alanine, glycine, or asparagine. The exchange of NAD^+^ and NADH via the malate valve is needed for this reaction, which is not the case if the reactions take place in the cytosol. Peroxisomal HPR was reported to be post-translationally modified by tyrosine nitration, which inactivated the enzyme. This suggest that the photorespiratory pathway is linked to peroxisomal NO metabolism (Corpas et al., 2013). Certain non-photosynthetic tissues, e.g., barley seed endosperm, contain little if any HPR-1 activity, but have considerable cytosolic HPR-2 activity (Igamberdiev and Kleczkowski, 2000), pointing out that its activity is not always necessarily linked to photorespiration. Depending on the metabolic status of the tissue, the enzyme may be involved in the pathway to serine formation from glycerate (via its reverse reaction), but may also, together with isocitrate dehydrogenase, contribute to the cytosolic NADPH/NADP^+^ turnover mechanism in this tissue. In the latter case, HPR-2 could utilize carbon skeletons derived from abundant amino acids in barley endosperm, including a substantial serine pool (Igamberdiev and Kleczkowski, 2000).

The cytosolic HPR-2 preferentially uses NADPH over NADH (Kleczkowski and Randall, 1988; Givan and Kleczkowski, 1992). In contrast to HPR-1, HPR-2 is inhibited by oxalate, tartronate and phosphohydroxypyruvate (Kleczkowski et al., 1990, 1991; Givan and Kleczkowski, 1992). It is also inhibited by supraoptimal concentrations of hydroxypyruvate (Kleczkowski and Randall, 1988; Givan and Kleczkowski, 1992) which may be important in regulation of serine biosynthesis when photorespiration is active. Unfortunately, its reaction in the direction of glycerate consumption and hydroxypyruvate formation was not studied extensively in plants. Nevertheless, analogous enzymes from different organisms have similar properties, therefore they can be useful references for this reaction. As an example, the NADP-dependent enzyme from the parasitic protist *Entamoeba histolytica* readily catalyzes the reaction of glycerate oxidation with lower affinity for glycerate than for hydroxypyruvate but higher maximal rates with glycerate than with hydroxypyruvate (Ali et al., 2003). Compared to HPR-2, even less is known about the HPR-3 enzyme, but its presence in the plastids (Kleczkowski et al., 1988; Timm et al., 2011) suggests that the glycerate pathway of serine synthesis can be active there, at least to some extent.

Interestingly, the pool of cytosolic glycerate used by HPR-2 may also serve as substrate for a cytosolic isozyme of glycerate kinase (GK). This enzyme was believed to be localized entirely in chloroplasts, where it constitutes the last step of the glycolate pathway (photorespiration) (Kleczkowski and Randall, 1985). However, recent studies on shade-grown Arabidopsis plants provided evidence for a cytosolic form of GK, in addition to the chloroplastic one, with both GK isozymes produced via a phytochrome-mediated alternative splicing of a single GK gene (Ushijima et al., 2017). Thus, whereas chloroplastic GK supplies the photorespiratory serine-derived carbon to the Calvin cycle, the cytosolic GK constitutes a cytoplasmic bypass of photorespiration, with its product 3-PGA entering glycolysis in the cytosol rather than the Calvin cycle in the chloroplasts (Ushijima et al., 2017). In this way, photorespiratory serine metabolism can be directly linked to glycolysis. Depending on whether HPR-2 or GK are using the cytosolic glycerate as substrate, the carbon flow is directed either toward serine synthesis (via glycerate – serine pathway) or glycolysis (via cytosolic GK).

Besides carrying reversible reactions, the different isozymes of plant HPRs may react with multiple substrates, in addition to hydroxypyruvate. For instance, both HPR-1 and HPR-2 are reactive with glyoxylate, producing glycolate (Kleczkowski and Randall, 1988). In addition, HPR-2 is also likely to use 4-hydroxyphenylpyruvate as substrate, producing D-4-hydroxyphenyllactate (pHPL), as shown for an enzyme from *Coleus blumei*, which has 76% identity (on amino acid basis) to Arabidopsis HPR-2 (Janiak et al., 2010; Hücherig and Petersen, 2013). pHPL is a precursor to rosmarinic acid, one of the most common caffeic acid esters found in plants. This would underlie a connection, at least at enzyme substrate specificity level, between serine metabolism and secondary compound formation. Plants contain also NAD(P)H-dependent glyoxylate reductases (GRs), which are practically inactive with hydroxypyruvate (Kleczkowski et al., 1986) but can reduce succinic semialdehyde (SSA) (see below) (Zarei et al., 2017).

Given the existence of several plant HPR and GR isozymes, which carry out reversible reactions and may differ in substrate specificity, enzymatic nomenclature of these proteins remains unsatisfactory and confusing. Enzyme nomenclature has the following entries: EC 1.1.1.26 (NADH-GR); EC 1.1.1.29 (NAD^+^-glycerate dehydrogenase); EC 1.1.1.79 (NADPH-GR, NADP^+^); EC 1.1.1.81 [NAD(P)H-hydroxypyruvate reductase]. Whereas cytosolic GR can be identified as EC 1.1.1.79 or, perhaps, EC 1.1.1.61 (defining its activity as SSA reductase/4-hydroxybutyrate dehydrogenase), the HPR-type enzymes (HPR-1, HPR-2, and HPR-3) should probably all be identified as EC 1.1.1.81. In this case EC 1.1.1.29 (glycerate dehydrogenase) is identical to EC 1.1.1.81, at least for HPR-1 which prefers NAD(H) over NADP(H). The exact nomenclature could only be clarified after systematic side-by-side comparison of substrate specificities of purified HPR and GR isozymes with reported substrates and their analogs as well as by comparison of amino acid sequences of these dehydrogenases/reductases. Also, in studies with plant extracts, different HPR and GR isozymes may be differentiated by the use of specific inhibitors (e.g., oxalate for HPR-2 or acetohydroxamate for cytosolic GR) (Givan and Kleczkowski, 1992).

### Serine Aminotransferase

Aminotransferases forming serine from hydroxypyruvate are also present outside of peroxisome, including cytosol and plastid (Timm et al., 2011). The reaction can proceed as alanine: hydroxypyruvate (serine: pyruvate) aminotransferase (EC 2.6.1.51) or glycine: hydroxypyruvate (serine: glyoxylate) aminotransferase (EC 2.6.1.45). The first reaction can link the classical glycolysis when pyruvate is converted to alanine with the glycerate pathway of serine synthesis, which reverts alanine for transamination of hydroxypyruvate. Usually aminotransferases exhibit broad specificity. It has been shown that Arabidopsis serine: glyoxylate aminotransferase (AGT1) is an asparagine aminotransferase (asparagine: oxo-acid transaminase; EC 2.6.1.14) having higher activity with asparagine (Zhang et al., 2013). It can transaminate hydroxypyruvate using alanine, asparagine, glycine and other amino acids.

## Phosphorylated (Phosphohydroxypyruvate) Serine Pathway

### Overview

While the glycerate pathway of serine synthesis is mainly cytosolic, the phosphorylated pathway (**Figure 1**) occurs in plastids. Its intermediate is phosphohydroxypyruvate instead of hydroxypyruvate, therefore phosphoserine is initially formed and three specific enzymes are involved: 3-PGA dehydrogenase (EC 1.1.1.95), phosphoserine aminotransferase (EC 2.6.1.52), and phosphoserine phosphatase (EC 3.1.3.3). An important study of the role of the phosphorylated pathway has been conducted on the double mutants of plastidial glyceraldehyde-3-phosphate dehydrogenase mutants (Anoman et al., 2015). While the phosphorylated serine biosynthesis links plastid glycolysis to amino acid metabolism, the branch of plastid glycolysis leading to pyruvate contributes to fatty acid biosynthesis in seeds (Flores-Tornero et al., 2017). It has been shown that 3-PGA pools between cytosol and plastid do not equilibrate in heterotrophic cells and that plastid glycolysis is metabolically separated from the cytosolic glycolysis (Flores-Tornero et al., 2017). The faster serine turnover during photorespiration caused by elevated activity of serine: glyoxylate aminotransferase progressively lowers day-time leaf serine contents and in turn induces the phosphoserine pathway (Modde et al., 2017). Serine biosynthesis via the phosphorylated pathway compensates for the lack of photorespiratory serine formation and provides 2-oxoglutarate for glutamate synthesis and for subsequent ammonium fixation (by the GS/GOGAT cycle) (Benstein et al., 2013).

### PGA Dehydrogenase

D-3-PGA dehydrogenase (PGDH) has been isolated, cloned and characterized in Arabidopsis (Ho et al., 1999b). The enzyme belongs to the NAD-dependent 2-hydroxyacid dehydrogenase family, and catalyzes a conversion of D-3-PGA to phosphoserine. Three genes for PGA-dehydrogenase have been found both in Arabidopsis and rice (Lasanthi-Kudahettige et al., 2007; Benstein et al., 2013; Toujani et al., 2013a,b). In mature plants, the most expressed gene is *PGDH1*, exhibiting the highest level of expression in the roots of light-grown plants which underlies its role in supplying serine to non-photosynthetic tissues, the genes *PGDH2* and *PGDH3* are only slightly expressed indicating their minor role in serine metabolism. A different pattern was observed in 10-day old seedlings where *PGDH3* was highly expressed only in cotyledons, while *PGDH2* was expressed in shoots, and *PGDH1* was expressed almost throughout the seedling (Benstein et al., 2013).

The enzyme exhibits the hyperbolic kinetics toward 3-PGA and NAD^+^ (Ho et al., 1999b; Benstein et al., 2013). All three forms of PGDH encoded by three corresponding genes in Arabidopsis are localized in plastids and possess similar kinetic properties, e.g., they exhibit very similar *K*m values with PGA and NAD^+^, *k*_cat_ and *V*_max_, however, they differ in the sensitivity to inhibition by serine (Benstein et al., 2013). The PGDH1 enzyme was inhibited at 5 mM concentration of serine, PGDH2 was unaffected by serine, and PGDH3 was most sensitive to serine being inhibited at its 1 mM concentration. The negative allosteric regulation of the two PGDH forms may be caused by binding of serine to the plant analog of the bacterial ACT domain, which was named after the bacterial enzymes aspartate kinase, chorismate mutase and TyrA (prephenate dehydrogenase) where it was first identified (Grant, 2006). Possible lower binding to such domain in PGDH1 and PGDH3 as compared to the ACT domain of the enzyme from *Escherichia Coli* where serine inhibition is observed already at 0.1 mM concentration, and the potential absence of this functional domain in PGDH2 needs further investigation. It is possible that PGDH2 participates not in serine but in 2-oxoglutarate biosynthesis that needs to proceed also at high concentrations of serine (Ros et al., 2014). The two isoforms of PGDH that are inhibited by serine, are allosterically activated by L-homocysteine at two orders of magnitude lower concentration than that of L-serine, indicating high regulatory potency of L-homocysteine for this enzyme (Okamura and Hirai, 2017).

In Arabidopsis, analyses of the T-DNA insertion null mutant *PGDH1* revealed an embryo-lethal phenotype, whereas PGDH-silenced lines obtained using a microRNA-based approach and having ∼40% amounts of PGDH compared to wild type plants, were inhibited in growth. Links between phosphoserine metabolism and tryptophan synthesis as well as ammonium assimilation were also observed, suggesting a vital function for PGDH for plant development and metabolism. The coexpression data indicate a possible function of the molecular form PGDH1 in tryptophan biosynthesis taking place via condensation of serine and indole, because *PGDH1* and the gene encoding phosphoserine aminotransferase (*PSAT1)* are strongly coexpressed with genes of tryptophan metabolism (Benstein et al., 2013). The lethality of *PGDH1* null mutant may be related to the impairment of biosynthesis of tryptophan and numerous secondary metabolites derived from this amino acid. In mammals, where PGDH controls the connection between glycolysis and serine formation, an increase in gene copy numbers for the enzyme, and thus its increased activity and increase in serine content, was linked to a variety of cancers (Kalhan and Hanson, 2012).

### Phosphoserine Aminotransferase

Phosphoserine aminotransferase (PSAT) or glutamate: phosphohydroxypyruvate (phosphoserine) aminotransferase uses glutamate for transamination (Ho et al., 1998). Under normal conditions, only minor PSAT activities can be detected in the leaves of spinach (Larsson and Albertsson, 1979) and pea (Walton and Woolhouse, 1986), while the enzyme was more active in the pea tissues associated with rapid cell proliferation, e.g., seeds, leaves and apical meristems (Cheung et al., 1968). In soybean root nodules, the enzyme (and the whole phosphoserine pathway) was proposed to be involved in purine biosynthesis for ureide production (Reynolds and Blevins, 1986).

Given that PSAT uses glutamate for transamination to form phosphoserine from phosphohydroxypyruvate, this means that it is connected with 2-oxoglutarate and glutamate metabolism. Since glutamate in stress conditions is decarboxylated to form GABA, the interference between the phosphorylated serine pathway and GABA shunt has been proposed (Anoman et al., 2015). It has been demonstrated that this aminotransferase is specific to the amino donor (glutamate). In the range of 5–50 mM, serine, threonine, valine, glycine, tryptophane, and *O*-acetyl-L-serine had no effect on the rate of reaction. Enzyme inhibition was observed with a high concentration of cysteine (Ho et al., 1998).

### Phosphoserine Phosphatase

Phosphoserine phosphatase (PSP) is inhibited by high concentrations of serine (Ho and Saito, 2001). It exhibits 1.5-fold abundance in the root compared with the leaf tissues (Ho et al., 1999a) and is induced under salinity stress (Purty et al., 2017). The enzyme was purified from Arabidopsis (Ho et al., 1999a), and its similarity to the mammal and bacterial enzyme has been demonstrated (Basurko et al., 1999). Mutants lacking PSP in Arabidopsis were embryo-lethal, with altered pollen and tapetum development (Benstein et al., 2013; Cascales-Miñana et al., 2013; Flores-Tornero et al., 2015), whereas overexpression of PSP resulted in the increase of leaf nitrate reductase activity in the light and increased photorespiration (Han et al., 2017). Mutant analyses revealed that the enzyme plays a crucial role in plant metabolism by affecting glycolysis, amino acid synthesis and the Krebs cycle (Cascales-Miñana et al., 2013).

## Metabolism of Serine Formed Via Non-Photorespiratory Pathways

### Formation of Glycine

**FIGURE 2:**
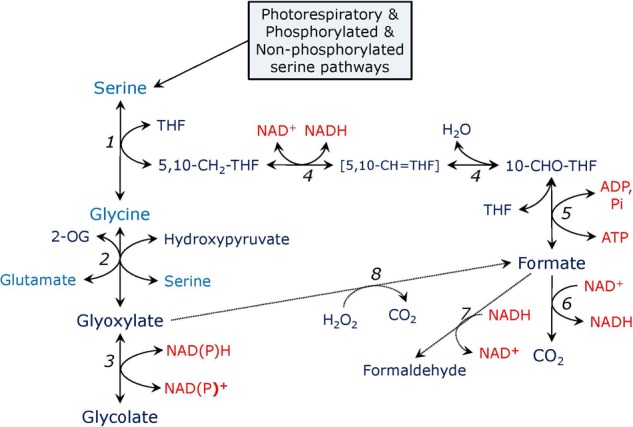
Pathways of serine metabolism. In the reaction catalyzed by serine hydroxymethyltransferase (1), serine is converted to glycine and 5,10-methylene-tetrahydrofolate (5,10-CH_2_-THF). Glycine is transaminated to glyoxylate by serine: glyoxylate or glutamate: glyoxylate aminotransferase (2). Glyoxylate is reduced to glycolate by glyoxylate reductase (3). 5,10-CH_2_-THF is converted to 10-formyl-tetrahydrofolate (10-CHO-THF) by the bifunctional enzyme methylenetetrahydrofolate dehydrogenase – methenyltetrahydrofolate cyclohydrolase (4), the intermediate is 5,10-methenyl-tetrahydrofolate (5,10-CH = THF). 10-CHO-THF is converted to formate and THF by formate-tetrahydrofolate ligase (5), the reaction yields ATP. Formate can be oxidized to CO_2_ by formate dehydrogenase (6) or reduced to formaldehyde by formaldehyde dehydrogenase which is also *S*-nitrosoglutathione reductase (alcohol dehydrogenase type III) (7). Formate can be formed from glyoxylate in a non-enzymatic reaction with hydrogen peroxide (8), glyoxylate in turn can be formed from formate in a putative glyoxylate synthetase reaction (not shown).

Serine formed in the glycerate and phosphorylated pathways can be further utilized in the reactions leading to glycine and formate production, as shown in **Figure 2**. Serine can be converted to glycine in the reaction carried out by serine hydroxymethyltransferase (SHMT). In Arabidopsis genome SHMT is encoded by seven genes, the products of two of which are targeted to mitochondria with SHM1 being the main mitochondrial isozyme (Voll et al., 2006), while SHM2 cannot replace it during photorespiration, being targeted to the vascular tissue (Engel et al., 2011), SHM3 is a plastidial form (Zhang et al., 2010), SHM4 and SHM5 are cytosolic, and SHM6 and SHM7 are located in the nucleus and their function is unknown (Bauwe and Kolukisaoglu, 2003). During photorespiration, SHMT operates in the direction of serine synthesis from glycine and 5,10-methylenetetrahydrofolate (5,10-CH_2_-THF) releasing tetrahydrofolate (THF), while in non-photosynthetic tissues, e.g., in roots, SHMT synthesizes glycine instead of consuming it (Ireland and Hiltz, 1995). The SHMT reaction can be presented as:

$$\begin{array}{c} L-Serine\;+\;\mathrm{THF}\;⇌\;\mathrm{Glycine}\;+\;5,{10-CH}_{2}-THF \end{array}$$

Arabidopsis mutants lacking or impaired in SHMT gene expression were smaller than the wild type plants, had less chlorophyll and accumulated hydrogen peroxide when subjected to either high light, high salt or to pathogen invasion (Moreno et al., 2005). The SHMT role in salt tolerance was also demonstrated for *Synechococcus elongatus* (cyanobacterium), where overexpression of SHMT resulted in enhanced salinity tolerance, accompanied also by increased activities of enzymes of the phosphorylated serine pathway as well as photorespiratory pathway (Waditee-Sirisattha et al., 2017).

The most straightforward utilization of glycine in heterotrophic tissues is its transamination forming glyoxylate. This reaction can be catalyzed by corresponding transaminases, which include glutamate: glyoxylate and serine: glyoxylate aminotransferases (Zhang et al., 2015). Glutamate: glyoxylate aminotransferase is a homolog of alanine: glyoxylate aminotransferase in Arabidopsis (Liepman and Olsen, 2003), and the activity is not necessarily associated with peroxisomes. Other aminotransferases may be also important including GABA transaminase which can operate with glyoxylate and GABA or glycine and SSA in reverse direction (Clark et al., 2009). The most active transaminase can be glutamate: glyoxylate (Zhang et al., 2015), which means that glycine transamination will form glutamate that can be further metabolized to GABA.

The fate of glyoxylate formed from glycine may be connected with its reduction to glycolate. GR activities (utilizing NADPH and NADH) in the cytosol (Givan and Kleczkowski, 1992) may result in lowering the redox level in the cell, which is particularly important under stress and may explain the observed increase of their activity (Igamberdiev et al., 1991). Their operation will result in the accumulation of glycolate, which has been identified as a stress compound. In non-photosynthetic tissues where glycolate cannot originate through photorespiratory metabolism, its most probable origin involves reduction of glyoxylate, resulting in efficient scavenging of this toxic compound. Another possible pathway of glyoxylate conversion is its condensation with succinate to form isocitrate (Eprintsev et al., 2015). The cytosolic form of isocitrate lyase, having low similarity with the Mg^2+^-dependent glyoxysomal form, operates at low pH, is Mn^2+^-dependent and may be efficient in operating under stress condition in the direction of isocitrate synthesis (Ivanov and Igamberdiev, 1989). This reaction may link serine pathway with GABA metabolism (see below). The links between citrate /isocitrate metabolism, serine and GABA formation have been established in metabolomics studies (Toubiana et al., 2016). Glyoxylate can be also taken as an alternative substrate for the plastidic pyruvate dehydrogenase complex (Blume et al., 2013) resulting in its decarboxylation.

### Serine as a Source of Formate

While one product of SHMT is glycine, another product is methylene-THF (**Figure 2**). The latter can be converted to formyl-THF by the bifunctional enzyme, which consists of methylenetetrahydrofolate dehydrogenase (EC 1.5.1.15) and methenyltetrahydrofolate cyclohydrolase (EC 3.5.4.9), and catalyzes the following two reactions:

(a) 5,10-CH_2_-THF + NAD^+^ ⇌ 5,10-CH = THF + NADH + H^+^
(b) 5,10-CH = THF + H_2_O ⇌ 10-CHO-THF

Based on genetic data, the bifunctional enzyme can be localized in mitochondria, in plastids and the cytosol (Ros et al., 2014); however, most of its activity was detected in the cytosolic fraction of pea leaves (Chen et al., 1997). Its product, formyl-THF, can release formate in the reaction catalyzed by 5,10-N-formyltetrahydrofolate synthetase, also named formate-tetrahydrofolate ligase (EC 6.3.4.3) (Nour and Rabinowitz, 1991):

$$\begin{array}{c} \mathrm{ADP}\;+\;\mathrm{Pi}\;+\;10-CHO-THF\;⇌\;\mathrm{ATP}\;+\;\mathrm{formate}\;+\;\mathrm{THF} \end{array}$$

The pool of formyl-THF as well as that of the ligase is mostly cytosolic (Iwai et al., 1967; Chen et al., 1997). The reaction may be of a special importance because it generates ATP which becomes a source of energy in the conditions of suppression of the electron transport chain in plant mitochondria. As a side product of SHMT, 5-formyltetrahydrofolate can be formed, which is inhibitory for SHMT and other C_1_- metabolizing enzymes, but it can be converted by 5-formyl-THF cycloligase (EC 6.3.3.2) to 5,10-methenyl-THF, and therefore it is a well-tolerated metabolite in Arabidopsis (Goyer et al., 2005).

Another possible way of releasing formate from formyl-THF is the reaction catalyzed by formyltetrahydrofolate deformylase (EC 3.5.1.10):

$$\begin{array}{c} 10-CHO-THF\;+\;H_{2}O\;⇌\;\mathrm{formate}\;+\;\mathrm{THF} \end{array}$$

This enzyme is present in plant mitochondria and participates in photorespiration (Collakova et al., 2008). Its role in the glycerate and phosphorylated pathways of serine formation is unlikely. The same refers to the reaction catalyzed by formyltetrahydrofolate dehydrogenase (EC 1.5.1.6), which presence in plants was not demonstrated:

$$\begin{array}{c} 10-CHO-THF\;+\;{\mathrm{NADP}}^{+}\;+\;H_{2}O\;⇌\;THF+\;{\mathrm{CO}}_{2}\;+\;\mathrm{NADPH}\;+\;H^{+} \end{array}$$

As it can be seen from the scheme of serine glycolysis, the pathway from 3-PGA to glycolate and formate results in the formation of NAD(P)H in PGDH reaction, and in methylene-THF dehydrogenase reaction, while NAD(P)H is scavenged in the GR reaction, and ATP is synthesized in formyl-THF synthetase reaction.

### The Fate of Formate and Glycolate

Glycolate can be formed as the end product in serine pathway under certain conditions, which include hypoxic stress. Under these conditions, the reduction of glyoxylate may be of benefit by decreasing the reducing power in the cell. A several-fold accumulation of glycolate was observed under conditions of oxygen deficiency in rice (Narsai et al., 2009). This can also take place in other stresses since the increase in redox level is a common phenomenon in stressed cells. The conversion of glyoxylate to glycolate can be achieved by several enzymes which include GRs. Some of them can also take hydroxypyruvate (Givan and Kleczkowski, 1992) or SSA (Brikis et al., 2017) as substrates. Conversion of glyoxylate to glycolate is achieved also by lactate dehydrogenase which has affinity also for glyoxylate. Some homologs of glycolate oxidase can convert lactate to pyruvate (Engqvist et al., 2015), while lactate dehydrogenase can reduce not only pyruvate but also glyoxylate (Mulcahy and O’Carra, 1997).

Further conversion of glycolate may be associated with glycolate oxidase which can be induced in stress conditions (Igamberdiev et al., 1991). Under oxygen deficiency glycolate oxidase cannot operate, but during reoxygenation it can utilize the glycolate pool accumulated under stress. The same refers to formate which oxidation can occur during post-stress period. The induction of formate dehydrogenase under different stresses including the hypoxic stress observed in plant species (Hourton-Cabassa et al., 1998) may be related to the necessity to utilize formate accumulated, in particular, from the reactions succeeding the phosphorylated and glycerate pathways of serine synthesis. A possibility of ATP synthesis during formate formation makes its accumulation an important mechanism for energy generation under stress. Besides the oxidation of formate by formate dehydrogenase, two molecules of formate can form glyoxylate in the condensation reaction (Janave et al., 1993). However, the enzyme carrying this reaction was characterized only once and its abundance in plants is not yet confirmed. Formate can be also converted to formaldehyde by formaldehyde dehydrogenase (EC 1.2.1.46) which is GSNO (nitrosoglutathione) reductase (Martinez et al., 1996). The enzyme is the alcohol dehydrogenase type III, it is induced under different stresses (Ticha et al., 2017), and it may link scavenging of reactive nitrogen species such as GSNO to formate metabolism.

The fate of formaldehyde needs further investigation, but it is known that it can be emitted by plants or converted to non-toxic compounds (Wang et al., 2016). Also, a recent study on trees has suggested that formaldehyde may represent an alternative carbon source for glycine methylation in photorespiration, and thus eliminating the need for a second glycine for the production of CH2-THF (Jardine et al., 2017).

### Link to GABA

**FIGURE 3:**
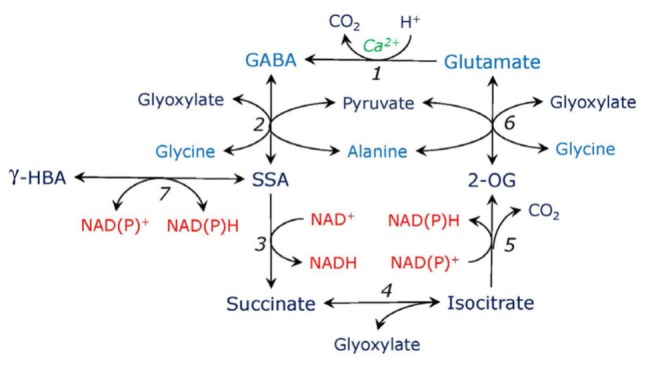
Putative γ-aminobutyrate-isocitrate cycle. Glutamate is decarboxylated by Ca-dependent glutamate decarboxylase (1), the reaction consumes proton and yields γ-aminobutyric acid (GABA). GABA is transaminated to succinic semialdehyde (SSA) by aminotransferases using glyoxylate or pyruvate (2). SSA is oxidized to succinate by SSA dehydrogenase (3). Succinate in the reaction with glyoxylate forms isocitrate, the reaction is catalyzed by the cytosolic form of isocitrate lyase (4). The latter is oxidized to 2-oxogutarate (2-OG) by isocitrate dehydrogenase (5). 2-OG is transaminated to glutamate by aminotransferases using glycine or alanine (6), use of other amino donors such as serine or aspartate is also possible (not shown). SSA can be converted to γ-hydroxybutyrate (γ-HBA) by SSA reductase which is also glyoxylate reductase (7).

An important consequence of the induction of the phosphorylated serine pathway caused by the introduction of double mutation in the plastidial glyceraldehyde phosphate dehydrogenase (Anoman et al., 2015) was activation of the expression of glutamate decarboxylase, the enzyme which forms GABA (**Figure 3**). It resulted in the increased levels of GABA in roots and in the aerial parts of Arabidopsis. This indicates a possibility of the link between GABA metabolism and the phosphorylated serine pathway. Accumulation of GABA prevented the buildup of glutamine in the double mutant of Arabidopsis, balanced carbon and nitrogen metabolism, and diverted anaplerotic carbon to the tricarboxylic acid cycle via SSA (Anoman et al., 2015). It has been shown that GABA synthesis and conversion can result in the shift in carbon-nitrogen balance (Fait et al., 2011). A more direct link between serine metabolism and GABA pathway may also exist, based on the fact that the label from glycine in stress conditions readily incorporates to GABA (Zemlyanukhin et al., 1980). This needs, however, further confirmation.

While the activity of glutamate decarboxylase takes place in the cytosol, the transamination of GABA that results in formation of SSA was considered to occur in mitochondria; however, it has been demonstrated that it can take place also in the cytosol (Cao et al., 2013). GABA transaminase can operate with glyoxylate and pyruvate but is inactive with 2-oxoglutarate (Clark et al., 2009); only a low activity of another transaminase taking 2-oxoglutarate was reported for some plants (Babu and Naik, 2013). A potential for interaction between GABA metabolism and photorespiratory glyoxylate production has been postulated (Clark et al., 2009), however, it is possible that the interaction may take place also with glyoxylate of the non-photorespiratory origin, e.g., originating from metabolism of serine formed in the glycerate and phosphorylated pathways.

An important observation has been made that SSA and glyoxylate are reduced by the same enzyme preferring NADPH over NADH (Clark et al., 2009). This enzyme can exist in two forms, one cytosolic (∼85% activity) and one plastidic and mitochondrial (∼15%), this has been shown for Arabidopsis, apple and rice (Brikis et al., 2017; Zarei et al., 2017). The cytosolic form is identical to the NAD(P)H-GR characterized by Kleczkowski et al. (1986) in spinach and by Kleczkowski and Edwards (1989) in maize. Since glyoxylate and SSA reduction can represent a mechanism of regulating redox level and detoxification of aldehydes during stress (Allan et al., 2009), the dual activity of these β-hydroxyacid dehydrogenases suggests a possible link between GABA and glyoxylate metabolism during stress. Identification of glycolate as a stress metabolite (Narsai et al., 2009) confirms this possibility.

Gamma-aminobutyric acid transamination with pyruvate yields alanine, which links GABA metabolism with the most common pathway of glycolysis. Operation of glycolysis with pyrophosphate under hypoxic conditions saves ATP and contributes to bioenergetics of the stressed cell (Igamberdiev and Kleczkowski, 2011). Formation of alanine diverts the glycolytic carbon to the neutral amino acid pool preventing acidification (as in the case of lactate) and loss of carbon (in the case of volatile ethanol), and the role of GABA in this process may be important. SSA formed in the reaction of GABA transamination can be either reduced to γ-hydroxybutyrate or oxidized to succinate. Although γ-hydroxybutyrate is likely a dead end product (as well as glycolate formed from glyoxylate by the same enzyme), its accumulation can decrease redox level of the stressed cell. Oxidation of SSA yields succinate which is accumulated in stress conditions. However, the enzyme forming succinate from SSA is more active at high pH (9-9.5) (Busch and Fromm, 1999), which makes its contribution under hypoxic and other stresses accompanied with cell acidification limited. It may be important when plant recovers from stress, e.g., during reoxygenation.

We propose that the GABA shunt (the pathway starting from 2-oxoglutarate and forming succinate which bypasses the TCA cycle) can be incorporated into a broader metabolic cycle operating in stress conditions, which is depicted on **Figure 3**. Succinate formed in the GABA shunt can be converted to isocitrate in the reaction catalyzed by the synthase reaction of cytosolic isocitrate lyase, which, in turn, is converted to 2-oxoglutarate by isocitrate dehydrogenase (preferentially its cytosolic form). In the stress conditions where aconitase is inhibited by reactive oxygen and nitrogen species (Gupta et al., 2012) and when the cytosolic isocitrate lyase is activated by lower pH and glyoxylate supply, this pathway can utilize glyoxylate and glycine from serine metabolism and contribute to pH regulation. SSA from the cycle can be diverted to formation of γ-hydroxybutyrate catalyzed by SSA/GR (Brikis et al., 2017), the reaction leading to decrease of redox level. While practically irreversible reaction of SSA conversion to succinate proceeds at high pH, it can utilize the γ-hydroxybutyrate pool formed under stress conditions.

### Other Conversions of Serine

Other pathways of serine conversions (except its direct incorporation into proteins) include its use in phospholipids and sphingolipids synthesis, synthesis (via one-carbon metabolism) of purines and pyrimidines, methionine and homocysteine.

## Non-Photorespiratory Metabolism of Serine and Environmental Stress

Serine may be a key player in biochemical responses of plants to environmental stress (Stewart and Larher, 1980). Serine accumulates at low temperatures (Draper, 1972), in hypoxic conditions (Guinn and Brinkerhoff, 1970), and in elevated salinity (Stewart and Larher, 1980). It is a precursor of ethanolamine, which is metabolized to glycine betaine which accumulates under stress (Rhodes et al., 1989). It was shown that the mRNA expression of PGDH increases under high salinity condition, whereas, flooding increases PSAT mRNA expression level (Ho and Saito, 2001; Lasanthi-Kudahettige et al., 2007). One gene of PGDH (LOC_Os04g55720.1) exhibited the fourfold expression in rice in anaerobic conditions, while two other were downregulated (Lasanthi-Kudahettige et al., 2007). The only gene of PSAT (LOC_Os03g06200.1) was upregulated by hypoxia fivefold in rice, while one gene of PSP was unchanged and another gene was downregulated twofold (Lasanthi-Kudahettige et al., 2007).

The role of serine in hypoxic stress has been established in the experiments using stable isotopes. The label from malate readily incorporated to serine and glycine in pea seedlings exposed to anoxia (Ivanov and Zemlyanukhin, 1991). Since malate can be connected to glycolysis via PEP carboxylase, it may deliver carbon to the phosphorylated and the glycerate pathways. The label from glycine goes readily to glycolate and GABA (Zemlyanukhin et al., 1980). Glycolate is formed from glyoxylate via the action of GR that can also reduce SSA (Brikis et al., 2017; Zarei et al., 2017). The GR activity increased twofold to threefold in rice and pea under anoxia, this effect was more prolonged in rice, while decarboxylation of glycine decreased under anoxia indicating that glycine was more readily utilized in other metabolic reactions (Igamberdiev et al., 1991). The gene of putative GR (NM_197517) was upregulated in rice threefold by oxygen deficiency (Lasanthi-Kudahettige et al., 2007). Glycolate was identified as an anaerobic metabolite in rice (Narsai et al., 2009), its accumulation increased many times during oxygen deficiency. Cryopreservation leading to hypoxic conditions also resulted in the activation of glycine and serine metabolism (Subbarayan et al., 2015). Similarly, in germinating seeds, which exhibit oxygen depletion (Narsai et al., 2017), serine metabolism may contribute to bioenergetics, and this needs further investigation. The ninefold accumulation of serine and activation of PGDH and PSAT has been reported in anaerobic rice coleoptiles (Shingaki-Wells et al., 2011). These responses may occur to a full extent in hypoxia-tolerant plants such as rice, while in Arabidopsis and other hypoxia-intolerant plants the observed changes in the levels of metabolites and enzymes are less prominent (Narsai et al., 2011; Narsai and Whelan, 2013).

GABA formation from glycine under low oxygen (Zemlyanukhin et al., 1980) needs further explanation. It indicates that the carbon from glycine can be readily incorporated to GABA, and since GABA precursor is glutamate, formation of the latter should be linked to glycine. The hypothesis has been suggested that glyoxylate (derived from glycine) can condense with succinate (accumulated under anoxia) via the synthase reaction of isocitrate lyase (Ivanov and Igamberdiev, 1989). The cytoplasmic form of this enzyme has low pH optimum (Eprintsev et al., 2015), which favors its operation under stress conditions when the cytosolic pH is decreased. Isocitrate formed in this reaction can be converted to 2-oxoglutarate and undergo the reactions leading to GABA biosynthesis.

To what extent the reactions of glycine and GABA formation under oxygen deficiency are linked to the activation of the phosphorylated and glycerate serine pathways needs further investigation. It is established that PSAT is induced under anoxia (Ho and Saito, 2001; Lasanthi-Kudahettige et al., 2007), however, two other enzymes of this pathway do not exhibit the increase in expression, at least PSP, while only one gene of PGDH is activated, two other suppressed (Lasanthi-Kudahettige et al., 2007). Nevertheless, the phosphorylated and glycerate serine pathways represent alternative versions of glycolysis and their role in anoxia needs further investigation.

NADH formed in the serine pathway and following reactions can be oxidized in the mitochondrial electron transport chain. In the case of oxygen deficiency or high load of the mitochondrial electron transport chain, nitrite can serve as an alternative electron acceptor (Gupta and Igamberdiev, 2011). It can be used by reduced hemeproteins and molybdocofactors yielding nitric oxide (NO). Nitrate reductase itself can use nitrite as a substrate forming NO. The induction of nitrate reductase during stress means activation of nitrogen metabolism, which is reflected in higher levels of major amino acids including GABA, serine, glycine, glutamate, etc. In the double nitrate reductase mutant of Arabidopsis, the hypoxic stress resulted in huge decrease in serine, glycine and GABA content, while in the wild type the levels of these amino acids were elevated in hypoxic conditions (Gupta et al., 2012). In Arabidopsis root cultures, two nitrate reductase genes were induced at low-oxygen (Klok et al., 2002). Although nitrite can be converted to NO, a significant part of nitrate converted by nitrate reductase turns to ammonium also in stress conditions when nitrite reductase is partially suppressed (Botrel et al., 1996; Ferrari and Varner, 1971). Ammonium ion from nitrate reduction facilitates glutamate synthesis either via the GS-GOGAT system consuming NADPH and ATP or via reverse glutamate dehydrogenase consuming only NAD(P)H (Gibbs and Greenway, 2003) which, in turn, stimulates formation of GABA.

The turnover of nitrate, nitrite and NO via the hemoglobin-NO oxide cycle in stress conditions (Igamberdiev and Hill, 2004; Gupta and Igamberdiev, 2011) involves only the oxidized forms of nitrogen, however, it maintains redox level in the cell and makes possible also the reactions of amino acid metabolism. Glutamate synthesis via the GS-GOGAT system requires ATP which can be supplied from the hemoglobin-NO cycle (Stoimenova et al., 2007) and in formate synthesis (Nour and Rabinowitz, 1991). Thus, serine/glycine metabolism becomes an important chain in NADH turnover in stressed cells that participates in energy homeostasis, pH regulation and ammonium fixation.

## Conclusion

The phosphorylated and glycerate-serine non-phosphorylated pathways of serine synthesis represent the branches of glycolysis. One operates in plastids and the other in cytosol. Both pathways can lead to the formation of glycolate and formate and connect metabolism with GABA shunt. Serine synthesis via the phosphorylated and non-phosphorylated pathways and its consecutive metabolism are important for the regulation of intracellular redox and energy levels and pH, in particular in stress conditions when the expression of several enzymes participating in these pathways is upregulated. This makes serine a key player in the biochemical adaptation to environmental stress. The pathway of serine conversion to formate is linked to substrate phosphorylation at the level of formate release.

## Author Contributions

Both the authors (AI and LK) have made substantial, direct and intellectual contribution to the work, and approved it for publication.

## Conflict of Interest Statement

The authors declare that the research was conducted in the absence of any commercial or financial relationships that could be construed as a potential conflict of interest.
