# Corrigendum: The Glycerate and Phosphorylated Pathways of Serine Synthesis in Plants: The Branches of Plant Glycolysis Linking Carbon and Nitrogen Metabolism

**DOI:** 10.3389/fpls.2018.00984

**Published:** 2018-07-04

**Authors:** Abir U. Igamberdiev, Leszek A. Kleczkowski

**Affiliations:** ^1^Department of Biology, Memorial University of Newfoundland, St. John's, NL, Canada; ^2^Department of Plant Physiology, Umeå Plant Science Centre, Umeå University, Umeå, Sweden

**Keywords:** glycerate serine pathway, phosphorylated serine pathway, γ-aminobutyric acid (GABA), plastid, glycolysis

In the original article, there was an error. In all mentions of hydroxybutyrate should be gamma (γ), not beta (β): γ-hydroxybutyrate.

This correction refers to the main text, to the legend of Figure 3, and to Figure 3 where β-HBA should be read as γ-HBA.

**Figure 3:**
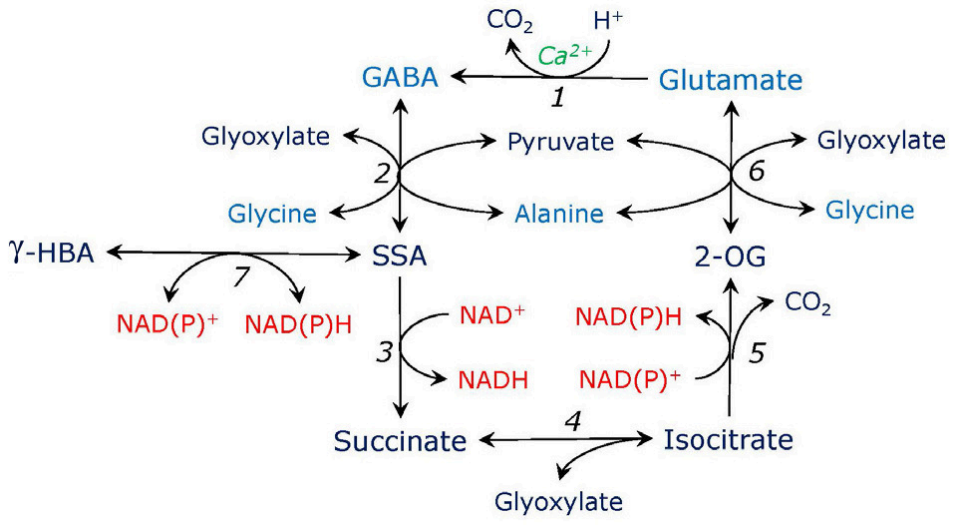
Putative γ-aminobutyrate-isocitrate cycle. Glutamate is decarboxylated by Ca-dependent glutamate decarboxylase (1), the reaction consumes proton and yields g-aminobutyric acid (GABA). GABA is transaminated to succinic semialdehyde (SSA) by aminotransferases using glyoxylate or pyruvate (2). SSA is oxidized to succinate by SSA dehydrogenase (3). Succinate in the reaction with glyoxylate forms isocitrate, the reaction is catalyzed by the cytosolic form of isocitrate lyase (4). The latter is oxidized to 2-oxogutarate (2-OG) by isocitrate dehydrogenase (5). 2-OG is transaminated to glutamate by aminotransferases using glycine or alanine (6), use of other amino donors such as serine or aspartate is also possible (not shown). SSA can be converted to γ-hydroxybutyrate (γ-HBA) by SSA reductase which is also glyoxylate reductase (7).

The authors apologize for this error and state that this does not change the scientific conclusions of the article in any way.

The original article has been updated.

## Conflict of interest statement

The authors declare that the research was conducted in the absence of any commercial or financial relationships that could be construed as a potential conflict of interest.

